# Supplementary material for: Novel application of ribonucleoprotein-mediated CRISPR-Cas9 gene editing in plant pathogenic oomycete species
Source: Microbiol Spectr. 2025 Feb 27;13(4):e03012-24. doi: 10.1128/spectrum.03012-24 (PMC11960053; doi:10.1128/spectrum.03012-24)
Supplement: File S1 — Supplemental methods, tables, and figures. [file spectrum.03012-24-s0004.pdf]

File S1: Supplementary Methods, Tables, and Figures

Supplementary Methods

Identification of the *PcORP1* orthologs in *Phytophthora ramorum* and *Phytophthora cactorum*

The protein, transcript, and genomic FASTA files for the *PcORP1* sequence were retrieved from the PhycoCosm server (1) using the Protein Id 564296 in the *Phytophthora capsici* LT1534 v11.0 genome (2). To identify the ortholog of *PcORP1* in *P. ramorum* (*PrORP1*), a tblastn (3) search was performed using the PcORP1 protein sequence as the query against the *P. ramorum* JGI reference genome (NCBI accession GCA_000149735.1, strain Pr102, lineage NA1). The top hit was selected, and the FASTA file for the corresponding genomic scaffold was downloaded. The genomic region corresponding to the sequence of the top hit from the tblastn search with an additional 1000 nucleotides of flanking upstream and downstream DNA was copied into Geneious Prime 2023.2.1 (https://www.geneious.com). The Geneious "Find ORFs" tool was used to analyze the genomic region and identify possible open reading frames (ORFs) in the sequence corresponding to the putative *PrORP1* gene. The longest ORF was selected and verified as an oxysterol-binding protein using the NCBI Open Reading Frame Finder (4) and SmartBLAST (5). Three independent pairs of primers were designed to amplify a region including the putative *PrORP1* ORF plus several hundred base pairs of flanking upstream and downstream DNA (Table S1). The same method was performed to find the *PcORP1* ortholog in *P. cactorum* (*PcaORP1*), but with the following minor modifications: the *P. cactorum* NCBI reference genome (NCBI accession GCA_016864655.1, strain P414) was used for the tblastn search of PcORP1, 1500 nucleotides of flanking upstream and downstream DNA were copied into Geneious for analysis, and only two primer pairs were designed to amplify the PcaORP1 region (Table S1).

Touchdown PCR of *PrORP1* and *PcaORP1* genes

Touchdown PCR was performed on DNA extractions from *P. ramorum* NA2_17 and *P. cactorum* FF42 to amplify approximately 3.5-4.8 kb regions of the *PrORP1* and *PcaORP1* genes, respectively. A high-fidelity polymerase, Phusion™ Plus DNA Polymerase (Thermo Fisher Scientific, Waltham, MA, USA), was used with the primers designed for each respective gene (Table S1). The PCR reactions were set up according to the manufacturer's instructions (Phusion™ Plus DNA Polymerase User Guide), and 5-10 ng of template DNA was used. The thermocycler conditions for touchdown PCR consisted of two phases; the cycle details are shown in Table S2. Phusion™ Plus DNA Polymerase uses a universal annealing temperature of 60°C, so this temperature was used for annealing in Phase 2 of the PCR.

Sanger sequencing and primer-walking for *PrORP1* and *PcaORP1*

Primer walking was performed along the touchdown PCR amplicons to determine the sequences of *PrORP1* and *PcaORP1* gene regions. For *PrORP1*, the PCR product from the Pr102ORPh_ORF_F1/R1 primers, named PrNA2ORPh, was sent for Sanger sequencing using the Pr102ORPh_ORF_F1 and Pr102ORPh_ORF_F2 primers for the forward direction, and the Pr102ORPh_ORF_R1 and Pr102ORPh_ORF_R3 primers for the reverse direction. The consensus sequences from the Sanger results were then used to design a new set of forward and reverse sequencing primers, which were used to sequence the next 500–700 base pairs of the original PrNA2ORPh PCR product. This process was repeated until the entire 3,891 bp PrNA2ORPh amplicon was sequenced. The same primer walking method was used for sequencing *PcaORP1*, using the 4,502 bp PCR amplicon from the Pca414ORPh_Seq_F1-A/R1-A primers, however, the primer walking was completed by the CHUL Sanger Sequencing Platform at Université Laval (CRCHU de Québec, Québec City, QC, Canada).

Gene prediction for *PrORP1* and *PcaORP1* sequences

The *PrORP1* and *PcaORP1* consensus sequences from primer walking were analyzed using the Geneious "Find ORFs" tool and the NCBI Open Reading Frame Finder (4) to identify putative ORFs. The largest ORF was selected and its protein sequence was analyzed with the SmartBLAST tool (5) on the NCBI Open Reading Frame Finder server to validate its identity as

an oxysterol-binding family protein. To predict the structure of each *ORP1* gene and find the exon-intron boundaries, the sequence of the putative ORF with approximately 500 nucleotides of flanking upstream and downstream DNA was analyzed using the Softberry FGENESH eukaryotic gene finding tool (http://www.softberry.com) with the *Phytophthora* gene-finding parameters selected. Softberry FGENESH was also used to analyze the *PrORP1* and *PcaORP1* ORFs and find their predicted proteins, PrORP1 and PcaORP1, respectively. The amino acid sequences of PrORP1 and PcaORP1 were analyzed with InterPro (6) to compare the predicted protein domains relative to the original *P. capsici* PcORP1 sequence (7).

Generation of single zoospore-derived cultures

Transformant cultures with *ORP1* mutations were taken through a single zoospore isolation protocol to ensure that downstream phenotyping would be performed on pure cultures derived from a mononucleate gene-edited zoospore. As mutations were only detected in *PcaORP1*, the protocol below outlines the method used for generating single zoospore cultures (SZCs) in *P. cactorum*, adapted from previous studies (8, 9). Wildtype *P. cactorum* FF42 cultures were also taken through the protocol to act as a control for downstream phenotyping. Cultures were grown on unamended V8A for two weeks. The agar on each plate was then cut into wedges with a sterile scalpel, flooded with 20 mL of autoclaved distilled water, and placed under continuous fluorescent light for 48 hours. The plates were then removed from the light and incubated at 4°C for 30 minutes, and the liquid from the plate was filtered through a 40 µm cell strainer into a 50 mL conical tube. Zoospore concentration was determined with a hemocytometer. The zoospore suspension was then diluted to the desired concentration, spread on a fresh V8A plate, and incubated in the dark at ambient temperature (approx. 21°C). After three to four days, each SZC was transferred to its own V8A plate. Three to four SZCs from each original *PcaORP1* mutant culture were taken through DNA extraction, PCR, Sanger sequencing, and TIDE analysis to confirm that the CRISPR-Cas9-mediated mutation was still present after single zoospore isolation.

Testing plasmid DNA retention in single zoospore cultures

To test whether the pYF2-PsCG plasmid DNA was retained in mutants after single zoospore isolation, PCR was performed on DNA from both the original *PcaORP1* mutant cultures and the corresponding SZCs. Phusion™ Plus DNA Polymerase (Thermo Fisher Scientific, Waltham, MA, USA) was used with the eGFP_Diag_F1/R1 primers designed to amplify a 273 base pair product from the eGFP gene on the pYF2-PsCG plasmid (10). Wildtype DNA from *P. cactorum* FF42 was used as the negative control, and plasmid DNA from pYF2-PsCG was used as the positive control. The PCR was performed with the same thermocycler conditions as described above, and the resulting PCR products were analyzed with gel electrophoresis.

Supplementary Tables

Table S1. Primer pairs designed to amplify the PcORP1 orthologs of *P. ramorum* (*PrORP*1) and *P. cactorum* (*PcaORP1*). All PCRs were performed using Phusion™ Plus DNA Polymerase, which creates a universal annealing temperature of 60°C.

<table>
<tr><th>Species
(Gene)</th><th>Primer Pair Name</th><th>Primer Sequences
(5’ to 3’)</th><th>Expected Product Size</th></tr>
<tr><td rowspan="6">Phytophthora ramorum (PrORP1)</td><td rowspan="2">Pr102ORPh_ORF_F1/R1</td><td>F1: CTGCGTCCAATCACAATATGCC</td><td rowspan="2">3,891 bp</td></tr>
<tr><td>R1: TCAAGCTGGCTCGTAACTTCAT</td></tr>
<tr><td rowspan="2">Pr102ORPh_ORF_F2/R2</td><td>F2: GTGTGACATTATTCCCAGTGGC</td><td rowspan="2">3,559 bp</td></tr>
<tr><td>R2: AGCTTCATTTACCCCACCAAGA</td></tr>
<tr><td rowspan="2">Pr102ORPh_ORF_F3/R3</td><td>F3: CTCATTTCGCCGGACAGTTG</td><td rowspan="2">3,485 bp</td></tr>
<tr><td>R3: ATGCTCATCCTGCTTTGCTTG</td></tr>
<tr><td rowspan="4">Phytophthora cactorum (PcaORP1)</td><td rowspan="2">Pca414ORPh_Seq_F1-A/R1-A</td><td>F1-A: CGATGGGGTTAGCACTACAGAG</td><td rowspan="2">4,502 bp</td></tr>
<tr><td>R1-A: CTTTAAGGAGAAACGCACGGAG</td></tr>
<tr><td rowspan="2">Pca414ORPh_Seq_F1-B/R1-B</td><td>F1-B: CTCTATCGCTCGTGTCTCTTCG</td><td rowspan="2">4,731 bp</td></tr>
<tr><td>R1-B: CCGGAGCTGAACCACTTCTACTTC</td></tr>
</table>

Table S2. Thermocycler conditions for touchdown PCR performed on *P. ramorum* NA2_17 and *P. cactorum* FF42 wildtype DNA using Pr102ORPh_ORF and Pca414ORPh_Seq primer pairs, respectively.

Phase	Step	Temperature	Time
	1	98°C	30 sec
1	2	98°C	10 sec
	3	70°C–1°C/cycle	15 sec
	4	72°C	30 sec/kb*
	**Repeat steps 2-4 for 10 cycles**		
2	5	98°C	10 sec
	6	60°C+	15 sec
	7	72°C	30 sec/kb*
	**Repeat steps 5-7 for 20 cycles**		
	8	72°C	5 min
	9	4°C	Hold

*Extension cycle is 30 seconds per kb of amplified product; 2 minutes was used for Pr102ORPh_ORF primers and 2.5 minutes was used for Pca414ORPh_Seq primers

+60°C is the universal annealing temperature for Phusion™ Plus DNA Polymerase

Table S3. Primer pairs designed to amplify CRISPR-Cas9 RNP-targeted regions of the *PrORP1* and *PcaORP1* genes of *Phytophthora ramorum* NA2_17 and *P. cactorum* FF42, respectively, for Sanger sequencing. The guide RNAs (gRNAs) that target a sequence within each primer-amplified region are also listed. A map of each gene with the locations of each primer and the respective gRNA-targeted regions is shown in Figure 1 of the main article. All PCRs for Sanger sequencing were performed using Phusion™ Plus DNA Polymerase, which uses a universal annealing temperature of 60°C.

Species (Gene)	Primer Pair Name	Primer Sequences (5' to 3')	gRNA(s) Amplified	Amplicon Size
Phytophthora ramorum NA2_17 (*PrORP1*)	PrNA2ORPh_F1/R1	F1: TTTTGCAGAAGAAAGGTCAGCG	Pr108rc<br>Pr192<br>Pr368mh	674 bp
		R1: CGCCTCCAAAACGTCCAGAC		
	PrNA2ORPh_F2/R2	F2: CCAAACGTACTTCTCGCATGAC	Pr751rc	740 bp
		R2: TTCCAGGATAAGCACCACAGAC		
Phytophthora cactorum FF42 (*PcaORP1*)	PcaORPh_Cas9Edit_1F/1R	1F: CGGATCTGGTGCATTTGTCAAG	Pca1272rcmh<br>Pca1507rcmh	766 bp
		1R: TCCTTGTCGATCAGCTTCAGAC		
	PcaORPh_Cas9Edit_2F/2R	2F: GAAGCCGTTTAACCCGATCTTG	Pca2248mh	571 bp
		2R: CATGTAGCCGCTCTGGAATTTG		
	PcaORPh_Cas9Edit_3F/3R	3F: ACCCTAGTGCAATCTTCCAAGC	Pca156<br>Pca330	777 bp
		3R: AAGCTCGTTTTCCAGTGCAATC		
	PcaORPh_Cas9Edit_4F/4R	4F: TACATGAAGGAGGGCTTCTTGC	Pca156	506 bp
		4R: CCATTAACCGTGGCAGTACTTG	Pca330	

Supplementary Figures

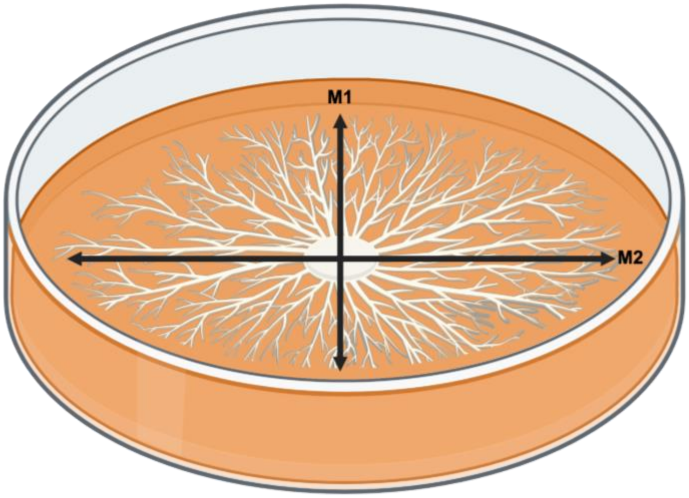

Figure S1. Schematic demonstrating how radial mycelial growth measurements were taken from *Phytophthora cactorum* cultures at seven days post plating on V8 agar medium supplemented with a gradient of oxathiapiprolin concentrations. To account for the width of the agar plug, 5 mm was subtracted from the length of measurement 1 (M1) and measurement 2 (M2). The average of the two measurements was recorded as the radial growth of each culture at a given oxathiapiprolin concentration. Created in BioRender. Dort, E. (2023) https://BioRender.com/s84r252.

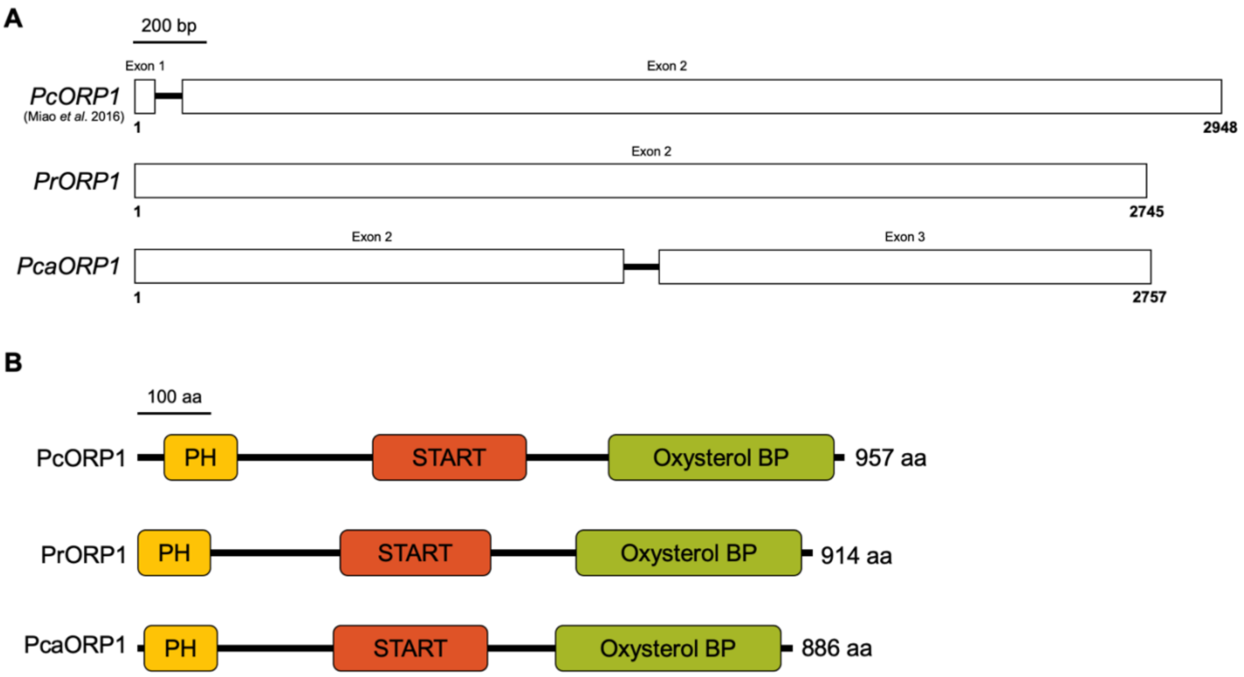

Figure S2. (A) Structure and size of the *PrORP1* and *PcaORP1* open reading frames relative to that of the *P. capsici PcORP1* gene characterized in 2016 by Miao et al. (7). The numbers indicate the size of the ORFs in base pairs (bp). Exon 1 is not shown for either *PrORP1* or *PcaORP1* as it was not predicted to be included in the ORF region of the genes. The exon-intron boundaries shown for *PcORP1* are according to Miao et al. (7). **(B)** Structure, size, and predicted functional domains of the PrORP1 and PcaORP1 proteins relative to the *P. capsici* PcORP1 protein characterized by Miao et al. (7). The amino acid sequences of all three proteins were analyzed using InterPro (6) to predict the functional domains.

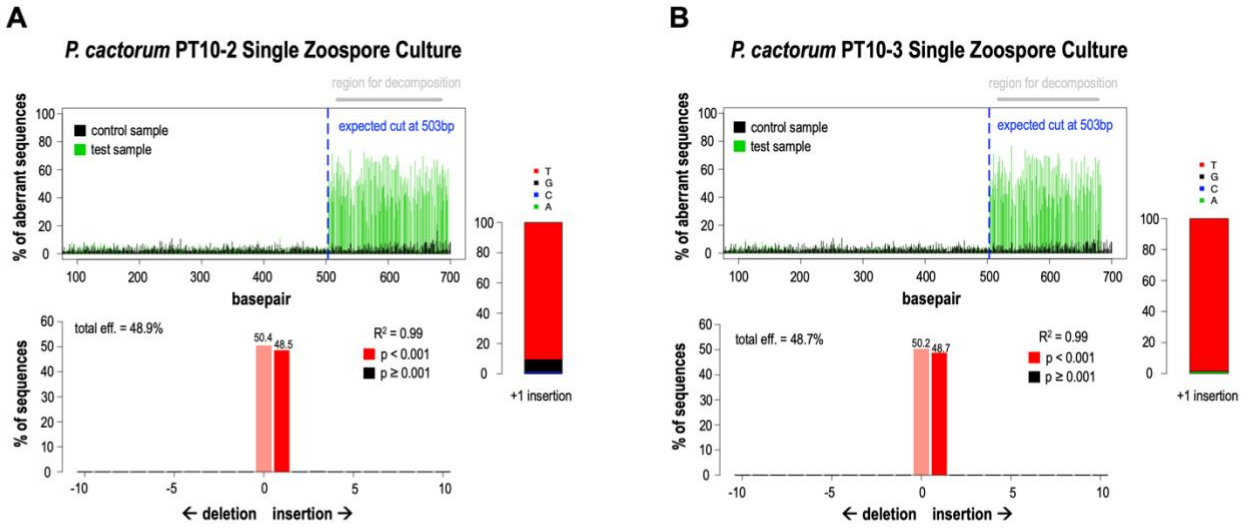

Figure S3. TIDE decomposition graphs for the remaining two single zoospore cultures (SZCs) of CRISPR-Cas9 *PcaORP1::1492* indel mutant PT10 (PT10-2 and PT10-3), obtained in *Phytophthora cactorum* FF42 using a plasmid-RNP co-transformation method with the Pca1507rcmh guide RNA (gRNA). The PT10 mutant is heterozygous, with the mutant allele comprising a single base pair insertion at the Cas9 cut site. The graphs shown were generated using TIDE software [Brinkman et al. 2014; (11)] on the forward Sanger sequencing results from PcaORPh_Cas9Edit_1F/1R PCR amplicons. The *P. cactorum PcaORP1* wildtype (WT) sequencing chromatogram was used as the control sample and the *PcaORP1::1492* PT10 mutant chromatograms were used as the test samples. The top graphs show the chromatogram sequence compositions with the expected Cas9 cut site indicated by the dashed blue line, the bottom graphs show the predicted percentage of sequences with wildtype vs. mutant genotypes, and the bar graphs to the right indicate the predicted nucleotide in the insertion mutants (y-axis represents probability). **(A)** TIDE decomposition graphs for PT10-2, the second SZC generated from PT10. The ratio of the wildtype to mutant DNA indicates a pure heterozygous culture with one wildtype allele and one mutant allele. **(B)** TIDE decomposition graphs for PT10-3, the third SZC generated from PT10. The ratio of the wildtype to mutant DNA indicates a pure heterozygous culture with one wildtype allele and one mutant allele. The TIDE results indicate that the nucleotide inserted on the sense strand of *PcaORP1* is a thymine, therefore the nucleotide inserted on the antisense strand where the gRNA was designed is an adenine.

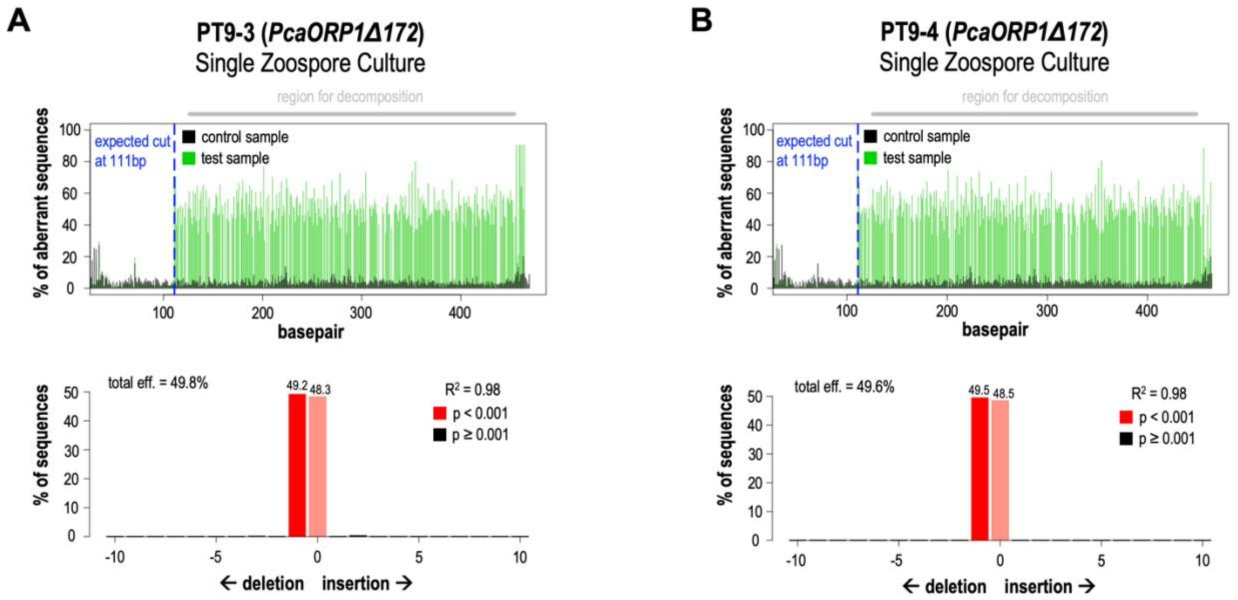

Figure S4. TIDE decomposition graphs for the remaining two single zoospore cultures (SZCs) of CRISPR-Cas9 *PcaORP1Δ172* indel mutant PT9 (PT9-3 and PT9-4), obtained in *Phytophthora cactorum* FF42 using a plasmid-RNP co-transformation method with the Pca156 guide RNA (gRNA). The PT9 mutant is heterozygous, with the mutant allele comprising a single base pair deletion at the Cas9 cut site. The graphs shown were generated using TIDE software [Brinkman et al. 2014; (11)] on the forward Sanger sequencing results from PcaORPh_Cas9Edit_4F/4R PCR amplicons. The *P. cactorum PcaORP1* wildtype (WT) sequencing chromatogram was used as the control sample and the *PcaORP1Δ172* PT9 mutant chromatograms were used as the test samples. The top graphs show the chromatogram sequence compositions with the expected Cas9 cut site indicated by the dashed blue line, and the bottom graphs show the predicted percentage of sequences with wildtype vs. mutant genotypes. **(A)** TIDE decomposition graphs for PT9-3, the third SZC generated from PT9. The ratio of the wildtype to mutant DNA indicates a pure heterozygous culture with one wildtype allele and one mutant allele. **(B)** TIDE decomposition graphs for PT9-4, the fourth SZC generated from PT9. The ratio of the wildtype to mutant DNA indicates a pure heterozygous culture with one wildtype allele and one mutant allele.

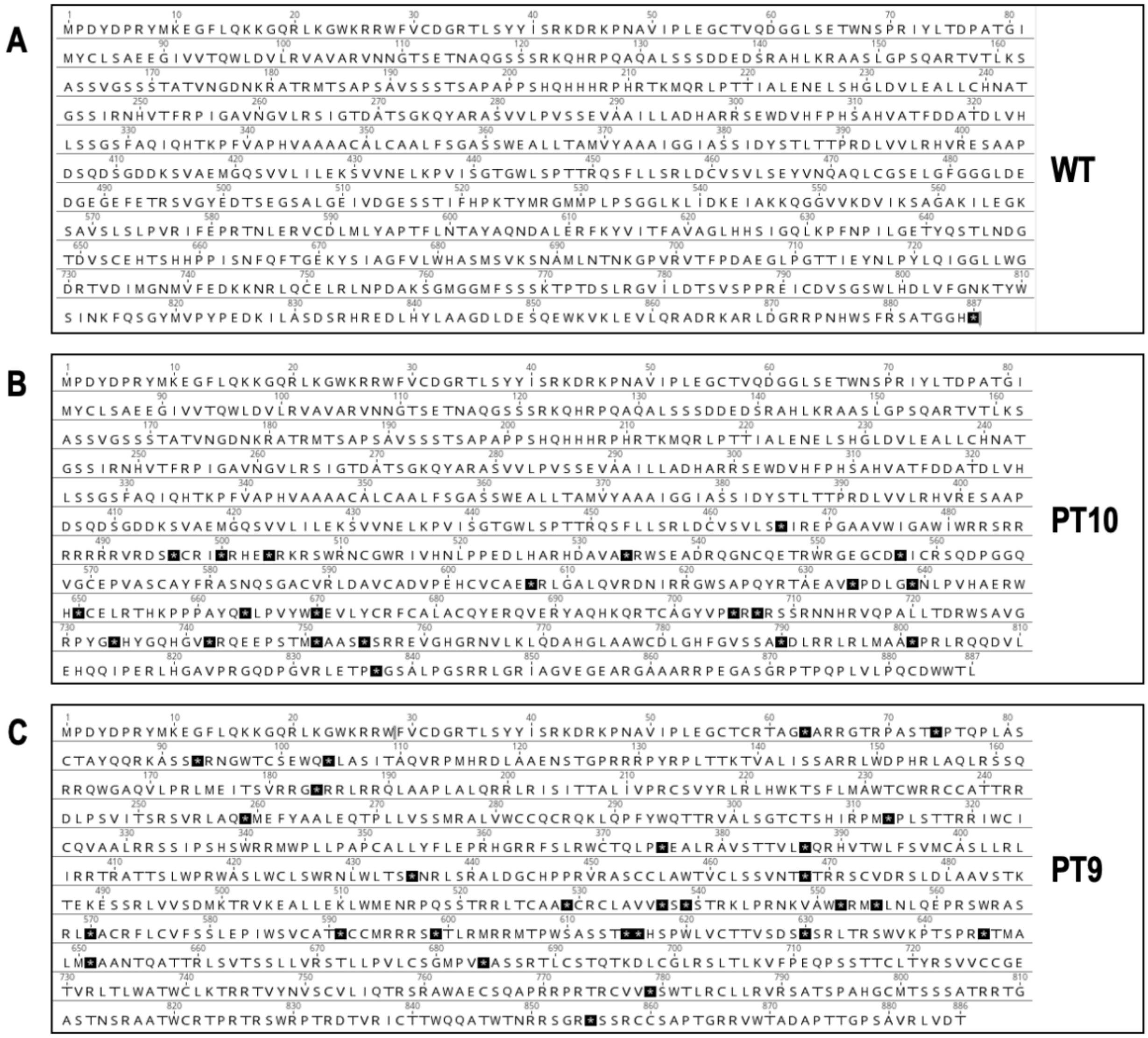

Figure S5. Predicted protein sequences for the *PcaORP1* gene of *Phytophthora cactorum* FF42 wildtype and mutant cultures. The black squares with asterisks indicate a stop codon. **(A)** The wildtype (WT) sequence encodes an 886 amino acid polypeptide. **(B)** The 1 base pair (bp) insertion mutation in one of the alleles of the *PcaORP1::1492* PT10 mutant introduces a frameshift resulting in a premature stop codon at amino acid 466. **(C)** The 1 bp deletion mutation in one of the alleles of the *PcaORP1Δ172* PT9 mutant also introduces a frameshift and results in a premature stop codon at amino acid 63. Protein sequences were generated using Geneious Prime 2023.2.1 (https://www.geneious.com).

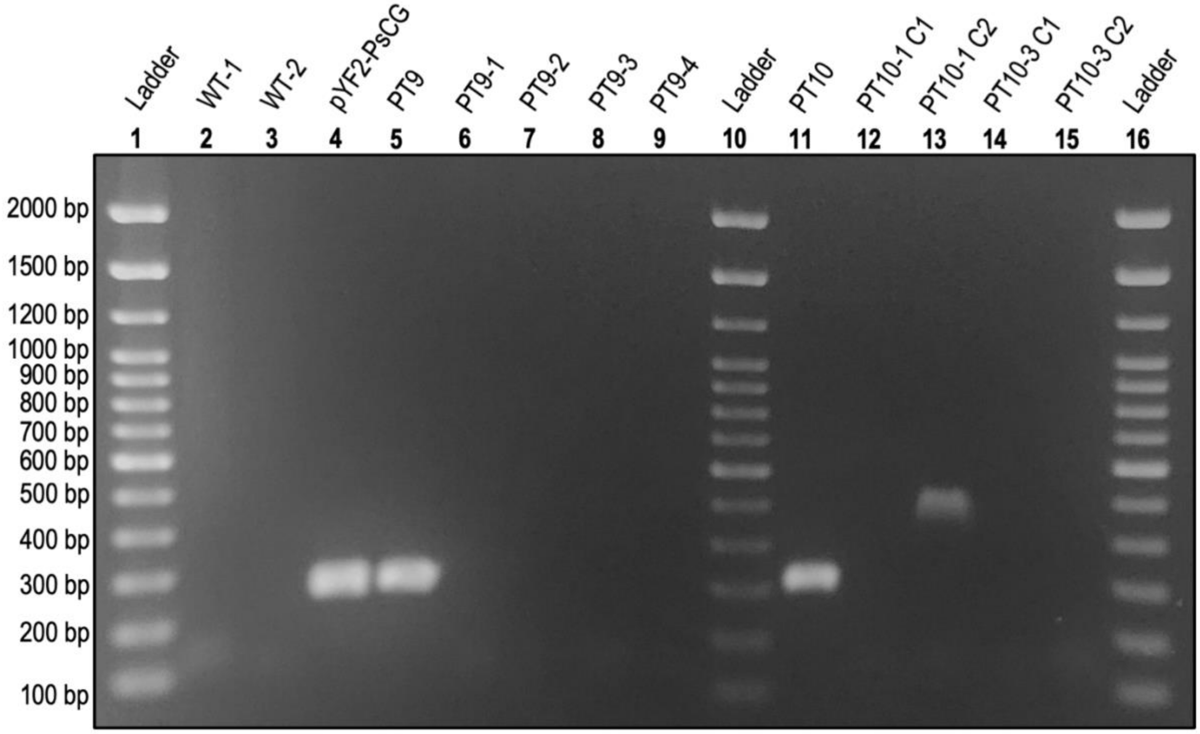

Figure S6. Agarose gel electrophoresis of products from a PCR performed using eGFP_Diag_F1/R1 primers (expected product = 274 bp) on DNA from *Phytophthora cactorum* FF42 *PcaORP1* mutant cultures (PT9, well 5; PT10, well 11) and their respective single zoospore cultures (SZCs): PT9-1 to PT9-4 (wells 6-9), PT10-1 (wells 12-13) and PT10-3 (wells 14-15). For the PT10 SZCs, DNA extractions from two replicate cultures were tested (C1, C2). The DNA from *P. cactorum* FF42 wildtype SZCs (WT-1 and WT-2) was used as the negative PCR control (wells 2-3), and pYF2-PsCG plasmid DNA was used as the positive PCR control (well 4).

References

1. Grigoriev IV, Hayes RD, Calhoun S, Kamel B, Wang A, Ahrendt S, Dusheyko S, Nikitin R, Mondo SJ, Salamov A, Shabalov I, Kuo A. 2021. PhycoCosm, a comparative algal genomics resource. Nucleic Acids Research 49:D1004–D1011.

2. Lamour KH, Mudge J, Gobena D, Hurtado-Gonzales OP, Schmutz J, Kuo A, Miller NA, Rice BJ, Raffaele S, Cano LM, Bharti AK, Donahoo RS, Finley S, Huitema E, Hulvey J, Platt D, Salamov A, Savidor A, Sharma R, Stam R, Storey D, Thines M, Win J, Haas BJ, Dinwiddie DL, Jenkins J, Knight JR, Affourtit JP, Han CS, Chertkov O, Lindquist EA, Detter C, Grigoriev IV, Kamoun S, Kingsmore SF. 2012. Genome Sequencing and Mapping Reveal Loss of Heterozygosity as a Mechanism for Rapid Adaptation in the Vegetable Pathogen *Phytophthora capsici*. MPMI 25:1350–1360.

3. Altschul SF, Gish W, Miller W, Myers EW, Lipman DJ. 1990. Basic local alignment search tool. Journal of Molecular Biology 215:403–410.

4. Wheeler DL, Church DM, Federhen S, Lash AE, Madden TL, Pontius JU, Schuler GD, Schriml LM, Sequeira E, Tatusova TA, Wagner L. 2003. Database resources of the National Center for Biotechnology. Nucleic Acids Res 31:28–33.

5. NCBI Resource Coordinators. 2016. Database resources of the National Center for Biotechnology Information. Nucleic Acids Research 44:D7–D19.

6. Paysan-Lafosse T, Blum M, Chuguransky S, Grego T, Pinto BL, Salazar GA, Bileschi ML, Bork P, Bridge A, Colwell L, Gough J, Haft DH, Letunić I, Marchler-Bauer A, Mi H, Natale DA, Orengo CA, Pandurangan AP, Rivoire C, Sigrist CJA, Sillitoe I, Thanki N, Thomas PD, Tosatto SCE, Wu CH, Bateman A. 2023. InterPro in 2022. Nucleic Acids Res 51:D418–D427.

7. Miao J, Cai M, Dong X, Liu L, Lin D, Zhang C, Pang Z, Liu X. 2016. Resistance Assessment for Oxathiapiprolin in *Phytophthora capsici* and the Detection of a Point Mutation (G769W) in PcORP1 that Confers Resistance. Frontiers in Microbiology 7:615.

8. Eikemo H, Stensvand A, Tronsmo AM. 2000. Evaluation of methods of screening strawberry cultivars for resistance to crown rot caused by *Phytophthora cactorum*. Annals of Applied Biology 137:237–244.

9. Eikemo H, Stensvand A, Davik J, Tronsmo AM. 2003. Resistance to crown rot (*Phytophthora cactorum*) in strawberry cultivars and in offspring from crosses between cultivars differing in susceptibility to the disease. Annals of Applied Biology 142:83–89.

10. Dort EN, Hamelin RC. 2024. Heterogeneity in establishment of polyethylene glycol-mediated plasmid transformations for five forest pathogenic *Phytophthora* species. PLOS ONE 19:e0306158.

11. Brinkman EK, Chen T, Amendola M, van Steensel B. 2014. Easy quantitative assessment of genome editing by sequence trace decomposition. Nucleic Acids Research 42:e168.
